# D-Dimer Measured at Diagnosis of Venous Thromboembolism is Associated with Risk of Major Bleeding

**DOI:** 10.1055/s-0039-1683395

**Published:** 2019-03-25

**Authors:** Håkon S. Johnsen, Kristian Hindberg, Esben Bjøri, Ellen E. Brodin, Sigrid K. Brækkan, Vânia M. Morelli, John-Bjarne Hansen

**Affiliations:** 1 Department of Clinical Medicine, K.G Jebsen – Thrombosis Research and Expertise Center (TREC), UiT - The Arctic University of Norway, Tromsø, Norway; 2 Division of Medicine, Akershus University Hospital, Lørenskog, Norway; 3 Division of Internal Medicine, University Hospital of North Norway, Tromsø, Norway

**Keywords:** venous thromboembolism, anticoagulants, major bleeding, D-dimer, biomarker

## Abstract

Identification of patients at risk of major bleeding is pivotal for optimal management of anticoagulant therapy in venous thromboembolism (VTE). Studies have suggested that D-dimer may predict major bleeding during anticoagulation; however, this is scarcely investigated in VTE patients. We aimed to investigate the role of D-dimer, measured at VTE diagnosis, as a predictive biomarker of major bleeding. The study population comprised 555 patients with a first community-acquired VTE (1994–2016), who were identified among participants from the Tromsø study. Major bleeding events were recorded during the first year after VTE and defined according to the criteria of the International Society on Thrombosis and Haemostasis. Cox-regression was used to calculate hazard ratios (HRs) with 95% confidence intervals (CIs) adjusted for age, sex, and duration of anticoagulant therapy. In total, 29 patients experienced major bleeding (incidence rate: 5.7/100 person-years, 95% CI: 4.0–8.2). The major bleeding risk was highest during the first 3 months, especially in patients with D-dimer ≥8.3 µg/mL (upper 20th percentile), with 28.8 major bleedings/100 person-years (95% CI: 13.7–60.4). Patients with D-dimer ≥8.3 µg/mL had a 2.6-fold (95% CI: 1.1–6.6) higher risk of major bleeding than patients with D-dimer ≤2.3 µg/mL (lower 40th percentile). Major bleeding risk according to D-dimer ≥8.3 versus ≤2.3 µg/mL was particularly pronounced among those with deep vein thrombosis (HR: 4.6, 95% CI: 1.3–16.2) and provoked events (HR: 4.2, 95% CI: 1.0–16.8). In conclusion, our results suggest that D-dimer measured at diagnosis may serve as a predictive biomarker of major bleeding after VTE, especially within the initial 3 months.

## Introduction

Anticoagulant therapy (AT) is the cornerstone in the treatment of venous thromboembolism (VTE). Extended AT effectively prevents recurrent events, but at the cost of bleeding complications. 1 2 3 The reported annual risk of major bleeding (MB) varies in the range of 1 to 4% 4 5 6 and is dependent on the choice of anticoagulant, intensity of anticoagulation, and duration of treatment. 6 7 8 The MB risk is particularly high within the first months of AT, 2 with a case-fatality rate of 11% during the initial 3 months of anticoagulation. 9

Even though initially promising in derivation studies, 10 11 12 prediction models developed to stratify risk of MB in VTE patients have demonstrated inconsistent discriminative powers in validation studies. 13 14 15 16 The existing prediction models mainly apply the same traditional predictors for bleeding such as age, history of bleeding, previous stroke, and cancer. 10 11 12 Identification of novel predictors for bleeding in VTE patients is therefore an essential step for the development of a more accurate prediction score for MB, capable of guiding clinical decision-making in the future.

D-dimer, a global biomarker of activation of the coagulation and fibrinolytic systems, is useful to exclude a VTE-diagnosis in the diagnostic work-up of suspected acute VTE. 17 Moreover, elevated D-dimer is used to identify patients at high risk of VTE-recurrence after discontinuation of AT. 18 19 Interestingly, elevated D-dimer levels have also been shown in conditions associated with increased bleeding risk, such as disseminated intravascular coagulation and acute abdominal aortic dissection. 20 21 Furthermore, high D-dimer levels appeared to predict MB during AT. 22 However, studies on VTE patients are scarce, 23 and whether D-dimer measured at VTE diagnosis can be used to assess risk of MB is largely unknown. Information on D-dimer at the time of VTE diagnosis is easily available for most patients with community-acquired VTE, as D-dimer is frequently used for the diagnostic work-up of VTE. 24 We aimed to investigate the role of D-dimer, measured at VTE diagnosis, as a predictive biomarker of MB events during the first year after an incident VTE.

## Methods

### Study Population

The source population comprised subjects participating in ≥1 of the six currently completed surveys of the Tromsø study (Tromsø 1–6), who were still alive and inhabitants of Tromsø by January 1, 1994 ( *n* = 33,885). The Tromsø study is a single-center, population-based prospective cohort, with repeated health surveys of the inhabitants of Tromsø, Norway. 25 Overall, participation rates were high, ranging from 85% in Tromsø 2 to 66% in Tromsø 6, with an average of 78.5% for the six surveys. The study was approved by the Regional Committee of Medical and Health Research Ethics, and all participants gave their informed written consent.

All potential first lifetime VTE cases were identified from January 1, 1994 to December 31, 2016 by searching the hospital discharge diagnosis registry, the autopsy registry, and the radiology procedure registry at the University Hospital of North Norway (UNN). The UNN is the only hospital serving the source population and all outpatient care for diagnostic assessment and treatment of VTE is exclusively provided at this hospital. The medical records of each potential VTE case were reviewed by trained personnel, and a VTE event was confirmed and registered as a validated VTE when clinical signs and symptoms of proximal or distal deep vein thrombosis (DVT) or pulmonary embolism (PE) were combined with objective confirmation by diagnostic procedures, and resulted in a VTE diagnosis requiring treatment, as described in detail previously. 26 Using the aforementioned strategy, a total of 986 objectively confirmed VTE cases were identified. D-dimer has low specificity for the diagnosis of VTE as it is often elevated in patients hospitalized for other conditions or with cancer. 27 28 29 We therefore excluded subjects with active cancer ( *n* = 230) and those already hospitalized for other conditions ( *n* = 108) when the VTE occurred. Moreover, subjects with high clinical suspicion leading to a VTE diagnosis without the aid of D-dimer measurement were also excluded ( *n* = 93), leaving 555 VTE patients eligible for this study. These patients were followed for 365 days, and all bleeding events occurring in this period were recorded by thorough review of medical records.

### Clinical Characteristics

Information on clinical and provoking factors at the time of and 12 weeks preceding the VTE diagnosis was obtained for all eligible patients. Patients with provoked VTE were those with major surgery, trauma, or an acute medical condition (acute myocardial infarction, ischemic stroke, or major infectious disease) within 12 weeks prior to VTE event, marked immobilization (confined to bed >3 days, wheelchair, or long-distance travel exceeding 4 hours within the last 14 days prior to VTE event), or any other factor specifically described in the medical records to have provoked the VTE (e.g., intravascular catheter).

Even though the study population was originated from a prospective cohort study (the Tromsø study), data collection for the present study was conducted retrospectively. To account for treatment duration in the present study, we considered the planned duration of anticoagulation that was objectively described by the attending physicians in the medical records at the time of VTE diagnosis. Duration of AT was categorized into 3, 6, and 12 months according to the preplanned length of AT. When the treatment duration was not specified by the treating physician ( *n* = 42), subjects were categorized into 3 months if the incident event was a provoked DVT, 6 months if it was an unprovoked DVT, and 12 months if it was a PE.

### Outcome Assessment of Major Bleeding

For each study participant, MB events occurring during the 365 days following the VTE were identified by thorough review of the medical records at the UNN. This hospital is the exclusive provider of advanced health care, including transfusion of blood products and emergency medicine in a vicinity of 250 km, and all subjects with a significant bleeding event in the Tromsø region are likely to be admitted at this hospital. Two reviewers (trained medical personnel from the UNN) adjudicated the bleeding events independently in accordance with the criteria proposed by the International Society on Thrombosis and Haemostasis. 30 In short, a bleeding event that was fatal, and/or symptomatic in a critical area or organ, and/or requiring blood transfusion of ≥2 units of red blood cells or causing a fall in hemoglobin level of ≥20 g/L was considered major. In case of disagreement, the event was discussed in an endpoint committee (H.S.J. and J.B.H.) to reach consensus.

### D-Dimer Measurement

Blood samples were drawn for the diagnostic work-up of VTE, before initiation of AT. D-dimer was determined using two commercially available kits at the Department of Clinical Chemistry at the UNN, 31 and a D-dimer value <0.5 μg/mL was defined as a negative test in the diagnostic work-up of patients with suspected VTE. The NycoCard D-dimer assay (Nycomed Pharma, Oslo, Norway), based on immunometric flow-through principle, was used in the period 1994 to 1998. It was succeeded by the STA-Liatest D-Di assay (Diagnostica Stago, Asnières-sur-Seine, France) for the remaining period (1998–2016). The Stago assay quantified D-dimer by the immuno-turbidimetric method (liquid reagent) within a range of 0.27 to 20 µg/mL, which determined the levels of D-dimer available in this study.

### Statistics

Subjects were followed from the date of their first VTE to the date of an incident MB, death, migration from Tromsø, or end of follow-up (i.e., 365 days after the first VTE), whichever came first. The patients were followed for 1 year, regardless of the length of anticoagulation. Thus, the follow-up time included both time-on and time-off anticoagulant treatment. Subjects who died or migrated were censored at the time of the respective event. Statistical analyses were performed with STATA version 15.0 MP (Stata Corp. College Station, Texas, United States).

D-dimer levels were initially divided into quintiles. The two lowest (Q1–2) and the two middle-upper (Q3–4) quintiles were combined to achieve better statistical power (i.e., a more robust reference category), and to enable the assessment of MB risk according to the highest D-dimer levels (Q5). The two lowest quintiles (Q1–2) were set as the reference.

Crude incidence rates (IRs) with 95% confidence intervals (CIs) of MB were calculated across D-dimer categories and expressed as number of events per 100 person-years at risk. Cox proportional hazards regression models were used to estimate hazard ratios (HRs) with 95% CIs for MB. The HRs were estimated using the following models: the first model was adjusted for age and sex, and the second was additionally adjusted for the planned duration of AT. Since a large severe thrombus could result in both high D-dimer levels and prolonged treatment, the treatment length was added as a potential confounder in the second model. The proportional hazards assumption was verified by evaluating the parallelism in the log–log survivor function by the categorical division of D-dimer. Further, the association between D-dimer levels and MB, adjusted for age, sex, and duration of AT, was visualized by a generalized additive regression plot using R version 3.4.4, to assess potential nonlinear effects of D-dimer levels on MB risk. D-dimer was modeled with a smoothing spline fit in a Cox proportional hazards model.

Due to potentially higher all-cause mortality rates in the upper D-dimer category, we additionally performed competing risk by death analyses and calculated subdistribution hazard ratios (SHRs) to limit overestimation of the relative risk differences of MB between D-dimer categories. 32 33 The 1-year cumulative incidences of MB across D-dimer categories were visualized in traditional one minus Kaplan–Meier (1-KM) plots and in cumulative incidence function plots corrected for competing risk by death.

We performed subgroup analyses stratified by clinical presentation (i.e., DVT and PE with or without DVT) and presence of provoking risk factors at the time of VTE diagnosis (i.e., unprovoked and provoked events). For overall VTE, we also assessed the risk of MB in analyses restricted to the first 3 months after VTE diagnosis (i.e., the period in which all patients would be on anticoagulant treatment). For sensitivity purposes, we performed analyses where we excluded patients who received thrombolytic therapy (systemic or catheter-directed) for VTE treatment, as these patients might be at increased bleeding risk. 3 We also did sensitivity analyses where patients were censored at the time they stopped anticoagulant treatment (estimated according to the planned duration of anticoagulation), to assess the risk of bleeding according to D-dimer restricted to the time on anticoagulant treatment.

## Results

Baseline characteristics according to D-dimer categories are shown in Table 1 . D-dimer levels were in the ranges of ≤2.3, 2.4 to 8.2, and ≥8.3 µg/mL in the lowest (Q1–2), middle (Q3–4), and upper (Q5) categories, respectively. The mean age and proportion of subjects with acute medical conditions preceding the VTE were higher in the upper than in the lower categories of D-dimer. Moreover, a higher proportion received thrombolytic therapy, and the planned duration of AT was longer in the highest category of D-dimer. The proportion of patients with PE was higher in the upper category, whereas DVTs were more frequent in the two lowest categories. Of note, missing information on duration of AT was similarly distributed across the lowest (7.6%), middle (7.3%), and upper (8.1%) D-dimer categories. The baseline characteristics of the overall study population can be found in Supplementary Table S1 .

**Table 1:** Baseline characteristics of venous thromboembolism (VTE) cases across categories of D-dimer

D-dimer, quintiles	Q1–2 ( *n* = 225)	Q3–4 ( *n* = 219)	Q5 ( *n* = 111)
Range (µg/mL)	≤2.3	2.4–8.2	≥8.3
Age (y), mean ± SD	64 ± 15	67 ± 14	69 ± 14
Sex (males)	44.0 (99)	56.2 (123)	50.5 (56)
Previous stroke	3.6 (8)	5.5 (12)	10.8 (12)
Thrombolytic therapy	1.8 (4)	6.9 (15)	10.8 (12)
Planned duration of anticoagulation			
≤ 3 mo	24.9 (56)	16.9 (37)	9.9 (11)
> 3 including 6 mo	43.6 (98)	48.0 (105)	38.7 (43)
> 6 including 12 mo	24.4 (55)	26.0 (57)	37.8 (42)
> 12 mo	7.1 (16)	9.1 (20)	13.5 (15)
VTE characteristics			
DVT	57.8 (130)	60.7 (133)	46.0 (51)
PE ± DVT	42.2 (95)	39.3 (86)	54.0 (60)
Unprovoked	64.0 (144)	59.8 (131)	60.4 (67)
Provoked	36.0 (81)	40.2 (88)	39.6 (44)
Trauma	12.0 (27)	10.0 (22)	10.8 (12)
Surgery	12.4 (28)	13.7 (30)	12.6 (14)
Acute medical condition	4.4 (10)	7.8 (17)	13.5 (15)
Confined to bed >3 days	1.8 (4)	1.8 (4)	2.7 (3)

Abbreviations: DVT, deep vein thrombosis; mo, months; PE, pulmonary embolism; SD, standard deviation.

Note: Categorical variables are shown as percentages with numbers in brackets, % ( *n* ).

Of the 555 patients with incident VTE, 29 had a MB event within 1 year after the incident VTE, yielding an overall IR of 5.7 per 100 person-years (95% CI: 4.0–8.2). The median and mean times from VTE diagnosis to MB were 35 and 113 days, respectively. MBs that were intramuscular with symptoms of compartment syndrome and gastrointestinal bleedings were most frequent (27.6%), followed by intracranial MBs (17.2%) ( Table 2 ).

**Table 2:** Sites of major bleeding (MB) in patients with venous thromboembolism

Bleeding site	MB, % ( *n* )
Intramuscular/compartment syndrome	27.6 (8)
Gastrointestinal	27.6 (8)
Intracranial	17.2 (5)
Urogenital	13.8 (4)
Other a	13.8 (4)

a Other sites of MB included pericardial, retroperitoneal, and subcutaneous (hematoma).

**Fig. 1:**
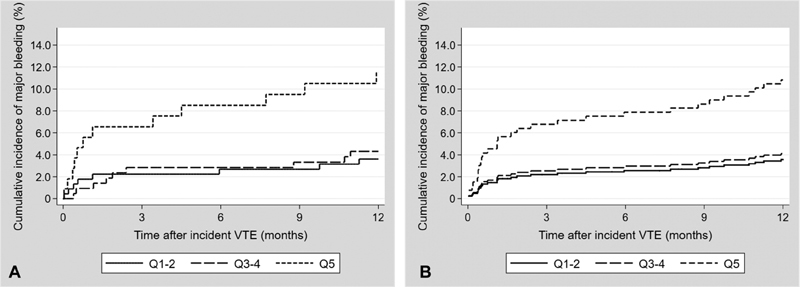
One year cumulative incidence of major bleeding by categories of D-dimer estimated by 1-Kaplan–Meier ( **A** ) and in the presence of death as competing event ( **B** ).

The 1-year cumulative incidences of MB across categories of D-dimer were estimated by 1-KM ( Fig. 1A ), and in the presence of death as competing risk ( Fig. 1B ), as displayed in Fig. 1 . The cumulative incidence of MB was considerably higher for the upper D-dimer category than for the lower and middle D-dimer categories ( Fig. 1A ). The results remained essentially similar after taking competing risk by death into account ( Fig. 1B ). The majority of the MB events occurred in the first 3 months after the VTE, and the 3-month cumulative incidences of MB were 2.2%, 2.5%, and 6.8% for patients in the lower, middle, and upper categories of D-dimer, respectively ( Fig. 1B ). At 12 months the cumulative incidences for the lower, middle, and upper categories were 3.6%, 4.1%, and 10.8%, respectively ( Fig. 1B ).

**Fig. 2:**
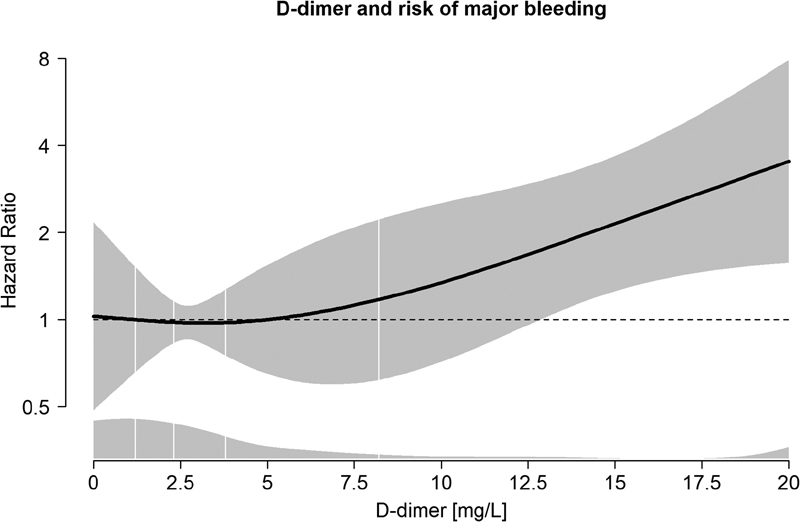
The risk of major bleeding (MB) as a function of D-dimer adjusted for age, sex, and planned treatment duration in a generalized additive regression model. The solid line shows hazard ratios (HRs), enclosed by shaded area showing 95% confidence intervals. The distribution of D-dimer is shown as density plots at the bottom, and in quintiles at the vertical lines.

The 3-month and 12-month IRs and HRs of MB in the overall population according to D-dimer categories are presented in Table 3 . At 12 months, the crude IR of MB was 13.1 per 100 person-years (95% CI: 7.5–23.1) in the upper category of D-dimer, versus 4.6 (95% CI: 2.4–8.9) and 3.8 (95% CI: 1.9–7.5) per 100 person-years in the middle and lower categories, respectively. In the model adjusted for age, sex, and duration of AT, patients with a D-dimer in the upper category (≥8.3 µg/mL) had a 2.6-fold higher risk of MB (HR: 2.6, 95% CI: 1.1–6.6) compared with those with a D-dimer in the lowest category (≤2.3 µg/mL). When analyses were restricted to the first 3 months of follow-up, the crude overall IR of MB was 13.9 per 100 person-years (95% CI: 8.8–22.1), and likewise the risk increased across categories of D-dimer, with an IR in the upper category of 28.8 per 100 person-years (95% CI: 13.7–60.4). Exclusion of patients who received thrombolytic therapy ( *n* = 31) yielded similar results as the main analyses ( Supplementary Table S2 ). The generalized additive regression plot revealed that the risk of MB started to increase at D-dimer levels >7.0 µg/mL ( Fig. 2 ).

**Table 3:** Incidence rates (IRs) and risk of major bleeding (MB) by categories of D-dimer at 12 and 3 months of follow-up after incident venous thromboembolism

12 mo	(D-dimer, µg/mL)	MB	IR (95% CI) a	HR (95% CI) b	HR (95% CI) c	SHR (95% CI) c
Q1–2	≤2.3	8	3.8 (1.9–7.5)	Ref.	Ref.	Ref.
Q3–4	2.4–8.2	9	4.6 (2.4–8.9)	1.1 (0.4–2.9)	1.1 (0.4–2.8)	1.0 (0.4–2.6)
Q5	≥8.3	12	13.1 (7.5–23.1)	2.9 (1.1–7.1)	2.6 (1.1–6.6)	2.5 (1.0–6.3)
**3 mo**						
Q1–2	≤2.3	5	9.3 (3.9–22.4)	Ref.	Ref.	Ref.
Q3–4	2.4–8.2	6	11.7 (5.3–26.1)	1.2 (0.4–3.9)	1.2 (0.4–3.9)	1.2 (0.4–3.8)
Q5	≥8.3	7	28.8 (13.7–60.4)	2.6 (0.8–8.4)	2.6 (0.8–8.6)	2.5 (0.7–8.8)

Abbreviations: CI, confidence interval; HR, hazard ratio; mo, months; SHR, subdistribution hazard ratio.

a Per 100 person-years.

b Adjusted for age and sex.

c Adjusted for age, sex, and planned duration of anticoagulation. SHR denotes the HR after taking competing risk by death into account.

Stratification according to specific subgroups (provoked, unprovoked, DVT, and PE) revealed that the association between D-dimer and MB was particularly pronounced among patients with DVTs and provoked events. In age-, sex-, and duration of AT-adjusted models, the HRs of MB according to D-dimer ≥8.3 versus ≤2.3 µg/mL were 4.6 (95% CI: 1.3–16.2) for patients with DVT and 4.2 (95% CI: 1.0–16.8) for those with provoked VTE ( Table 4 ). In contrast, a D-dimer ≥8.3 µg/mL was associated with a marginally increased risk of MB in patients with PE (HR: 1.7, 95% CI: 0.4–6.9) or unprovoked events (HR: 1.5, 95% CI: 0.4–5.9), but the results were not statistically significant. As in the overall analyses ( Table 3 ), the results were only slightly attenuated in the competing risk model ( Table 4 ), as demonstrated by the SHRs.

**Table 4:** Incidence rates (IRs) and risk of major bleeding (MB) by categories of D-dimer in subgroups of venous thromboembolism

DVT ( *n* = 314)	(D-dimer, µg/mL)	MB	IR (95% CI) a	HR (95% CI) b	HR (95% CI) c	SHR (95% CI) c
Q1–2	≤2.3	4	3.2 (1.2–8.5)	Ref.	Ref.	Ref.
Q3–4	2.4–8.2	6	4.9 (2.2–10.9)	1.5 (0.4–5.3)	1.1 (0.3–4.1)	1.1 (0.3–3.9)
Q5	≥8.3	8	19.9 (10.0–39.8)	5.0 (1.4–17.7)	4.6 (1.3–16.2)	4.4 (1.1–18.1)
** PE ( *n* = 241) **						
Q1–2	≤2.3	4	4.5 (1.7–11.9)	Ref.	Ref.	Ref.
Q3–4	2.4–8.2	3	3.8 (1.2–11.8)	0.8 (0.2–3.6)	0.8 (0.2–3.6)	0.8 (0.2–3.3)
Q5	≥8.3	4	7.5 (2.8–20.0)	1.7 (0.4–6.8)	1.7 (0.4–6.9)	1.5 (0.3–7.8)
** Unprovoked ( *n* = 342) **						
Q1–2	≤2.3	5	3.6 (1.5–8.7)	Ref.	Ref.	Ref.
Q3–4	2.4–8.2	5	4.1 (1.7–9.9)	1.1 (0.3–3.9)	1.1 (0.3–3.9)	1.1 (0.3–3.7)
Q5	≥8.3	4	6.7 (2.5–17.8)	1.7 (0.5–6.5)	1.5 (0.4–5.9)	1.5 (0.3–6.1)
** Provoked ( *n* = 213) **						
Q1–2	≤2.3	3	3.9 (1.3–12.2)	Ref.	Ref.	Ref.
Q3–4	2.4–8.7	4	5.0 (1.9–13.2)	1.0 (0.2–4.5)	0.8 (0.2–3.7)	0.8 (0.2–3.5)
Q5	≥8.8	8	23.9 (12.0–47.8)	4.2 (1.0–16.9)	4.2 (1.0–16.8)	3.6 (0.9–14.8)

Abbreviations: CI, confidence interval; DVT, deep vein thrombosis; HR, hazard ratio; PE, pulmonary embolism; SHR, subdistribution hazard ratio.

a Per 100 person-years.

b Adjusted for age and sex.

c Adjusted for age, sex, and planned duration of anticoagulation. SHR denotes the HR after taking competing risk by death into account.

Among the 29 MB events, only one case occurred after the preplanned treatment length. Sensitivity analyses restricted to the time on anticoagulant treatment showed essentially similar results ( Supplementary Table S3 and Supplementary Fig. S1 ).

## Discussion

In this population-based cohort study of patients with a first lifetime community-acquired VTE, we found that patients with high D-dimer levels (≥8.3 µg/mL) had 2.6-fold higher risk of a MB during the 1 year of follow-up compared with those with D-dimer levels ≤2.3 µg/mL. The risk of MB was highest during the first few months after the VTE. In patients with high D-dimer, the 3-month cumulative incidence of MB was 6.8%, whereas the cumulative incidence for the entire 12-month follow-up was 10.8%. The risk of MB among patients with D-dimer ≥8.3 µg/mL was especially high for those with DVTs and provoked events. Our findings suggest that a high D-dimer value at VTE diagnosis identifies patients at increased risk of MB events, particularly in the initial phase of anticoagulant treatment.

In the present study, the overall 1-year IR of MB was 5.7 per 100 person-years, which is comparable to previously reported rates of MB in patients treated with warfarin. 34 Consistent with previous data, 2 we found that the IR of MB was notably high during the first 3 months after VTE, especially in those with high D-dimer (28.8 MBs per 100 person-years). Possible explanations for the initially high bleeding risk may include overanticoagulation due to the wide intra- and interindividual variability in the dose-requirements in patients treated with vitamin K antagonists (VKAs), 35 36 and the initial administration of concomitant low molecular weight heparin. Furthermore, patients with a bleeding predisposition are more likely to experience a MB early after initiation of AT. 3

Our results are consistent with previous studies on atrial fibrillation, 37 38 such as the ARISTOTLE-trial, 37 where patients with a D-dimer ≥1,123 µg/L had a twofold increased risk of MB compared with those with a D-dimer <423 µg/L. To date, only a few studies, with substantial differences in designs and sample sizes, have examined the association of D-dimer with risk of MB in VTE patients. 22 23 In line with our results, a study of 1,707 PE patients from the RIETE-registry found that a D-dimer ≥4.2 µg/mL was associated with increased risk of MB within 15 days of PE diagnosis. 23 In a study comprising 719 patients treated with VKA for at least 2 months before inclusion, of whom only 11% had VTE, Lind et al found that high D-dimer levels, measured during AT, were associated with increased risk of MB. 22 To the best of our knowledge, the present study is the first to provide data on the association between D-dimer and risk of MB in a cohort of community-acquired VTE, encompassing both DVT and PE, without active cancer at the time of VTE diagnosis.

In our study, the risk of MB in patients with high D-dimer levels was most pronounced among those with provoked events. It is reasonable to assume an overestimation of the MB risk for comorbidities with high mortality rates. 33 39 40 However, the risk estimates for MB remained essentially similar when the competing risk of death was taken into account. Hence, our findings suggest that the association between high D-dimer and MB risk in subjects with provoked VTE could not be explained by an overestimation due to high mortality rates. Still, other medical conditions associated with increased risk of provoked DVT, but not necessarily with higher mortality within the first year after an incident VTE, could be relevant for the association between D-dimer and MB. 29 41

Several studies have shown that elevated D-dimer is associated with increased risk of recurrence in patients with unprovoked VTE. 18 19 Using data from the Tromsø study, we recently reported that a low D-dimer (≤1.5 µg/mL), measured at first VTE diagnosis, was associated with a low recurrence risk, particularly among patients with DVTs and unprovoked events. 31 In the present study, we found that a high D-dimer, also measured at VTE diagnosis, was associated with risk of MB. Intuitively, for the purpose of discriminating between VTE patients at high risk of either MB or recurrent VTE, such a predictive factor may appear noninformative, as D-dimer is associated in the same direction for both outcomes. However, the risk of MB and recurrence seemed to be most pronounced in different parts of the spectrum of D-dimer values. Only the very high levels of D-dimer (>7.0 µg/mL) were predictive of MB, whereas the recurrence risk did not further increase for D-dimer levels >1.5 µg/mL (threshold effect). 31 Moreover, the risk of MB and recurrence displayed different patterns in cumulative incidence curves. For recurrence, the risk gradually increased over years in patients with a D-dimer ≥1.5 µg/mL, 31 whereas for MB, the risk rapidly increased within the first 3 months after the incident VTE in patients with a D-dimer ≥8.3 µg/mL.

Our findings have potential clinical implications for the management of VTE during anticoagulation. D-dimer may be used as a predictive biomarker for MB to guide decisions on duration of AT, particularly in combination with clinical predictors of MB. 13 Importantly, this may be achieved without the need for additional blood sampling or cost as D-dimer is measured at VTE diagnosis in most patients. Given that the absolute risk and potential to prevent MB is highest during the first few months of anticoagulation, D-dimer may have clinical utility for the short-term management of VTE. Improved assessment of individual bleeding risk may impact clinical management, such as the choice of anticoagulant drug, careful supervision of anticoagulation, prompt investigation of minor gastrointestinal or urogenital bleeding to eliminate possible sources of future MB, or avoidance of concomitant therapies that may cause bleeding (e.g., antiplatelet agents and nonsteroidal anti-inflammatory drugs). 42 Moreover, after a first unprovoked event, identification of patients at high risk of bleeding is of utmost importance for decisions on extended duration of AT. 43 Even though we found relatively weak associations between D-dimer levels and risk of MB in patients with unprovoked events, the potential of D-dimer as a contributing building block in a prediction model in this specific patient group remains to be determined. Finally, during the study period, the majority of the patients in our study were treated with VKAs, as direct oral anticoagulants (DOACs) became available for clinical practice in Norway around 2012. Therefore, future studies are needed to confirm if D-dimer also is a predictive biomarker of bleeding in patients treated with DOACs.

The inclusion of subjects derived from the general population is among the main strengths of the present study. In contrast, bleeding complications are often studied as a safety outcome in randomized clinical trials, which tend to include selected patients compared with those from population-based studies, whose clinical characteristics more likely reflect real-life patients. Other strengths include the prospective design, complete and validated registry of VTE events, and the exclusivity of UNN as the sole health care provider, likely to receive all relevant MB events. The study also has some limitations. Our results are not generalizable to patients already hospitalized for other conditions or with active cancer when the VTE occurred. For this patient group, however, D-dimer may already be of limited clinical utility given its reduced specificity in the diagnostic work-up of VTE. 27 28 Even though the UNN is the sole health care provider within a geographically well-defined region, we cannot rule out the unlikely possibility that a MB event was not captured due to the retrospective collection of data. In this study, we did not have access to information on the actual duration of anticoagulant treatment, and we based our adjustments on the preplanned treatment length. This could have led to misclassification of treatment length in some patients. Nevertheless, when we restricted our analyses to the first 3 months after VTE diagnosis (i.e., the period in which all patients would be on anticoagulant treatment), the results were essentially similar to the overall analyses, showing an increased risk of MB associated with a high D-dimer value (≥8.3 µg/mL). Unfortunately, we did not have information on the concomitant use of drugs that might affect the bleeding risk, such as the use of antiplatelet medication. Approximately 14% of eligible patients were excluded because of missing D-dimer values. Of these, there were three MB events, yielding an IR of 3.5 per 100 person-years (95% CI: 1.1–10.9), which was lower compared with the rate of the included population. However, this rate was based on few MBs, and clinical characteristics were not widely different in those with measured and missing D-dimer (data not shown). Taken together, missing values of D-dimer was presumably at random, and would unlikely introduce selection bias. We used two different assays to measure D-dimer levels, which might have led to misclassification due to varying analytical properties across D-dimer assays. 44 However, the STA-Liatest, which has consistently reported excellent analytical properties, 45 46 was used for 93.5% of the study population, thus limiting misclassification. Moreover, in sensitivity analysis restricted to subjects with D-dimer determined by the STA-Liatest, the results remained essentially the same (data not shown). Finally, our results should be interpreted with caution due to low numbers of MB events and limited statistical power, mainly in subgroup analyses.

In conclusion, our findings suggest that high levels of D-dimer (≥ 8.3 µg/mL), measured at the time of first VTE diagnosis, identify patients at increased risk of MB, particularly during the first 3 months of AT. Future studies are warranted to confirm our findings and to investigate whether D-dimer at VTE diagnosis could improve risk stratification of MB when added to existing prediction models.
