# Supplementary material for: D-Dimer Measured at Diagnosis of Venous Thromboembolism is Associated with Risk of Major Bleeding
Source: TH Open. 2019 Mar 25;3(1):e77–84. doi: 10.1055/s-0039-1683395 (PMC6524911; doi:10.1055/s-0039-1683395)
Supplement: Supplementary file 1 — Supplementary Material [file 10-1055-s-0039-1683395-s180063.pdf]

Supplementary Table S1 Baseline characteristics of the overall population

Number of patients	555
Age (y), mean ± SD	66 ± 15
Sex (males)	50.0 (278)
Previous stroke	5.8 (32)
Thrombolytic therapy	6.6 (31)
Duration of anticoagulation	
≤ 3 mo	18.7 (104)
> 3 including 6 mo	44.3 (246)
> 6 including 12 mo	27.8 (154)
> 12 mo	9.2 (51)
VTE characteristics	
DVT	56.6 (314)
PE ± DVT	43.4 (241)
Unprovoked	61.6 (342)
Provoked	38.4 (213)
Trauma	11.0 (61)
Surgery	13.0 (72)
Acute medical condition	7.6 (42)
Confined to bed >3 days	2.0 (11)

Abbreviations: DVT, deep vein thrombosis; mo, months; PE, pulmonary embolism; SD, standard deviation.
Note: Categorical variables are shown as percentages with numbers in brackets, % (*n*).

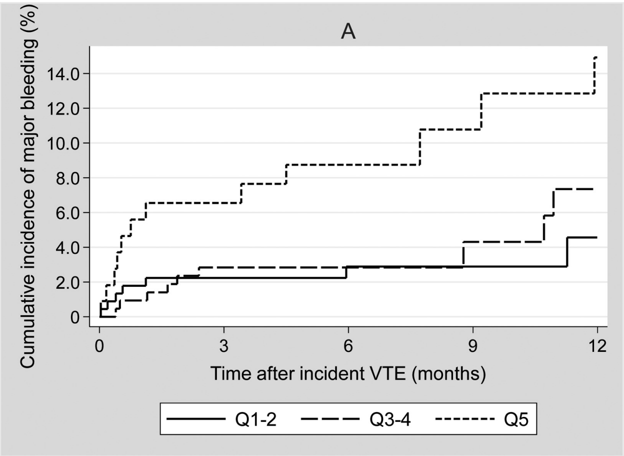

Supplementary Fig. S1 One year cumulative incidence of major bleeding by categories of D-dimer estimated by 1-Kaplan–Meier with follow-up restricted to time on anticoagulant therapy.

Supplementary Table S2 Incidence rates (IRs) and risk of major bleeding (MB) by categories of D-dimer after incident venous thromboembolism, with the exclusion of patients who received thrombolytic treatment (*n* = 31)

Overall (*n* = 524)	(D-dimer, µg/mL)	MB	IR (95% CI)[a]	HR (95% CI)[b]	HR (95% CI)[c]	SHR (95% CI)[c]
Q1–2	≤2.3	8	3.8 (1.9–7.6)	Ref.	Ref.	Ref.
Q3–4	2.4–8.2	9	4.8 (2.5–9.2)	1.2 (0.4–3.0)	1.1 (0.4–3.0)	1.1 (0.4–2.8)
Q5	≥8.3	10	11.8 (6.4–22.0)	2.6 (1.0–6.7)	2.4 (0.9–6.3)	2.2 (0.8–6.0)

Abbreviations: CI, confidence interval; HR, hazard ratio; SHR, subdistribution hazard ratio.
[a]Per 100 person-years.
[b]Adjusted for age and sex.
[c]Adjusted for age, sex, and planned duration of anticoagulation.

Supplementary Table S3 Incidence rates (IRs) and risk of major bleeding (MB) by categories of D-dimer with follow-up restricted to time on anticoagulant therapy

	(D-dimer, µg/mL)	MB	IR (95% CI)[a]	HR (95% CI)[b]	SHR (95% CI)[b]
Q1–2	≤2.3	7	5.7 (2.7–12.1)	Ref.	Ref.
Q3–4	2.4–8.2	9	7.2 (3.7–13.8)	1.2 (0.4–3.3)	1.2 (0.5–3.2)
Q5	≥8.3	12	17.8 (10.1–31.4)	2.9 (1.1–7.4)	2.7 (1.0–7.1)

Abbreviations: CI, confidence intervals; HR, hazard ratio; SHR, subdistribution hazard ratio.
Note: Time on anticoagulant therapy was determined according to the planned duration of anticoagulation described in the medical records at venous thromboembolism diagnosis.
[a]Per 100 person-years.
[b]Adjusted for age and sex.
